# Postmarket Drug Surveillance Without Trial Costs: Discovery of Adverse Drug Reactions Through Large-Scale Analysis of Web Search Queries

**DOI:** 10.2196/jmir.2614

**Published:** 2013-06-18

**Authors:** Elad Yom-Tov, Evgeniy Gabrilovich

**Affiliations:** ^1^Yahoo ResearchNew York, NYUnited States

**Keywords:** machine learning, information retrieval, side effects, infoveillance, infodemiology

## Abstract

**Background:**

Postmarket drug safety surveillance largely depends on spontaneous reports by patients and health care providers; hence, less common adverse drug reactions—especially those caused by long-term exposure, multidrug treatments, or those specific to special populations—often elude discovery.

**Objective:**

Here we propose a low cost, fully automated method for continuous monitoring of adverse drug reactions in single drugs and in combinations thereof, and demonstrate the discovery of heretofore-unknown ones.

**Methods:**

We used aggregated search data of large populations of Internet users to extract information related to drugs and adverse reactions to them, and correlated these data over time. We further extended our method to identify adverse reactions to combinations of drugs.

**Results:**

We validated our method by showing high correlations of our findings with known adverse drug reactions (ADRs). However, although acute early-onset drug reactions are more likely to be reported to regulatory agencies, we show that less acute later-onset ones are better captured in Web search queries.

**Conclusions:**

Our method is advantageous in identifying previously unknown adverse drug reactions. These ADRs should be considered as candidates for further scrutiny by medical regulatory authorities, for example, through phase 4 trials.

## Introduction

Existing mechanisms for postmarket drug surveillance work well in many cases, but failures resulting in harm to patients and even fatalities are widely documented [1], including the withdrawal of thalidomide in the 1960s [2], and more recently of cerivastatin [3], troglitazone [4], and rofecoxib [5]. Two main kinds of postmarket drug surveillance mechanisms exist today. One kind is run by regulatory agencies, such as MedWatch by the US Food and Drug Administration (FDA) and the Vaccine Adverse Event Reporting System (VAERS) by the FDA and the Centers for Disease Control and Prevention (CDC) in the United States, the Yellow Card Scheme by the Medicines and Healthcare products Regulatory Agency (MHRA) in the United Kingdom, and the International Drug Monitoring Programme by the World Health Organization (WHO). These are supplemented by public (or public-private cooperation) initiatives, such as Research on Adverse Drug Events and Reports (RADAR), and Web sites, such as eHealthMe.com, which collect patient-reported information on drug outcomes. The most serious limitation of these data collection initiatives is that they rely on the patients and their health care providers to make the association between the adverse drug reaction (ADR) and the drug. This can be especially difficult when the adverse reaction appears only after the drug is taken for a lengthy period of time, or when the patient takes several medications concurrently. To alleviate this problem, projects such as the FDA’s Sentinel Initiative [6], the EU-ADR initiative [7], and the Observational Medical Outcomes Partnership (OMOP) [8-11] are beginning to use observational data, including administrative claims and electronic health records, to identify adverse drug reactions.

Our proposed approach uses a novel kind of observational data, namely, Web search query logs. Search queries contain a cornucopia of world knowledge, and prior studies have used query logs to track tropical storms [12], certain life events [13], and the spread of disease [14]. As such, this approach is an example of infodemiology [15], and is enabled by the fact that as many as 80% of US Internet users seek health information online [16]. Consequently, our methodology allows analyzing the data from literally hundreds of millions of people, and in some cases, a significant percentage of the patients using a given drug. Performing such analysis continually allows for long-term monitoring, whereas grouping search requests by geographical location facilitates demographic segmentation of the population [17].

Existing drug surveillance mechanisms often depend on the need for medical providers or patients to realize the connection between the treatment and its side effects (adverse or otherwise). This inherent limitation poses a challenge to testing new methods for ADR discovery because existing data are not comprehensive enough to be considered a gold standard, considering that patients and medical providers might not realize the connection between treatments and some ADRs. Therefore, we adopted a 2-pronged approach for validating our method. First, we showed that it can reliably identify currently known ADRs. Although the findings of our method are positively correlated with existing data, this correlation is not perfect as we discover new, previously unknown ADRs. Second, we characterized the differences between the known and the newly discovered ADRs, and identified the most discordant ADRs (MDADRs) between these 2 sources. We show that the ADRs found by our method are usually less acute reactions (ie, not requiring immediate medical attention) with much later onset, which is exactly why they elude detection by conventional mechanisms. For all the drugs examined, we found that the ADRs apnea and cramps are consistently overlooked in the FDA data, as reported in the Adverse Event Reporting System (AERS), described subsequently, whereas tiredness and weight loss are frequent ADRs of vaccines that are overlooked in VAERS reports. We propose that the ADRs newly discovered by our method be further investigated in carefully designed clinical trials, which should be lengthy enough to allow detection of late-onset reactions.

## Methods

Our method, called the query log reaction score (QLRS), quantifies the prevalence of ADRs for a given drug, as explained subsequently. We used QLRS to identify ADRs of top-selling drugs and vaccines on the basis of queries submitted to the Yahoo US Web search engine during 6 months in 2010. A total of 176 million unique users, as identified by a unique signature of the users’ browser, were included in this study. The search logs were anonymized according to the Yahoo privacy policy by scrambling actual user identifiers. This was achieved by using a 1-way cryptographic hash function, which makes it impossible to map the resultant hash values back to the original user identifiers, while keeping the probability of collisions very low. As explained subsequently, only the search counts were considered, which were aggregated across users. Furthermore, the research described herein was carried out according to the Yahoo guidelines on human subject research.

We investigated 20 drugs (additional results for the top 100 drugs are provided in Multimedia Appendix 1), which are the top-selling drugs in the United States by revenue [18]. We analyzed these drugs for 2 reasons. First, these findings would likely affect the largest number of people. Second, data are more abundant for these drugs; thus, results are likely to be more significant for these drugs. We note that all these drugs are usually taken for long periods of time; however, we have also demonstrated the applicability of our method to vaccines, as detailed in Multimedia Appendix 1, which are usually administered a limited number of times to each patient. We limited our work to nongeneric versions of these drugs to reduce the chance of additional confounding influences, and because brand names are mentioned 88% more often than generic names in the query log (not statistically significant). However, we also discuss the differences in ADRs of similar drugs in the Results section.

A total of 195 symptoms from *The International Statistical Classification of Diseases and Related Health Problems* 10th Revision (ICD-10) [19] were studied as manifestations of possible ADRs. We filtered the symptoms according to Wikipedia’s List of Medical Symptoms [20] to facilitate replicability of our method in other languages. This list of symptoms was further expanded with synonyms (described subsequently), because patients frequently use nonmedical terminology to describe their symptoms. Basing our work on terms from Wikipedia (a popular information source) and identifying synonyms using behavioral data makes our approach suitable for identifying ADRs as described by nonprofessionals.

We limited the symptoms under consideration for each drug to the 50 most frequently queried symptoms for that drug. We identified possible ways nonprofessionals described their health symptoms by using 2 query expansion methods. First, we selected the most frequent search terms that led users to click on the Wikipedia page that described each symptom [21-23]. Second, we extracted frequently occurring lexical affinities [24], namely, word pairs appearing in close proximity in the 50 highest ranked Web search results returned when the symptom name was used as a query. The 2 top terms from each of the 2 methods were used as alternative names for each symptom. For each symptom discussed in the paper, the various possible search terms expressing it have been mapped to the same medical term. For example, the ADR *diplopia* could have been searched for by using the colloquial term *double vision*.

**Table 1:** The chi-square contingency table for a given drug and adverse drug reactions (ADRs) used for computing the query log reaction score (QLRS).

When user queried for the ADR	User queried for the drug?	
	No	Yes
Before Day 0	N11	N12
After Day 0	N21	N22

Three medical professionals (2 medical doctors, 1 nurse practitioner) independently labeled the expansion terms with respect to their relevance as an expansion term to each specific medical term. The interannotator agreement estimated using the Fleiss’ kappa statistic [25] was 0.44 (*P*<.001). This is a medium-level agreement. However, for 88% of the terms, most annotators (ie, ≥2) agreed that the term was an appropriate expansion of the medical term. Thus, our expansion method constructs a high-precision dictionary of terms. To maintain the automated nature of our method, the results reported here are based on all the expansion terms, not just those marked as relevant by the annotators.

For each drug, we first identified all the users who had searched for the drug name. For those, we define Day Zero for each user as the day when that user first searched for the drug. Day Zero for all other users (who did not search for the drug) was defined as the midpoint of their observed query history. We then counted the number of times each symptom was queried before and after Day Zero by each user. The purpose of using the data from people who did not search for the drug was to normalize against environmental effects, eg, seasonal allergies. This is in contrast with most prior infoveillance research [15], which is concerned with whole-population prevalence rather than the comparison of specific subpopulations, eg, people using or not using the drug.

For each drug-symptom pair, we constructed a 2-way contingency table counting the number of times a symptom was searched for before and after Day Zero, for users who did and did not search for the drug (see Table 1). For each symptom, we scored its prevalence as a reaction to the drug using the Pearson’s goodness of fit test, the chi-square test statistic [26]. We refer to this score as the query log reaction score (QLRS). Additional results for the top 100 drugs are provided in Multimedia Appendix 1.

We used 2 reference datasets to assess the validity of our findings. Adverse Event Reporting System (AERS, currently known as the FDA Adverse Event Reporting System) is the database of the FDA’s postmarket safety surveillance program for approved pharmaceutical drugs. The Side Effect Resource (SIDER) lists known ADRs for marketed drugs, extracted from public documents and package inserts [27].

The AERS data were downloaded from the FDA AERS website [28], and included reports submitted between January 2004 and June 2010. Reports were mapped to the same list of symptoms as QLRS using the same synonym list. In total, 47% of the cases in AERS were matched to at least 1 of the 195 symptoms or their synonyms, indicating good coverage by the symptoms list used in our study. Similar analysis was performed for SIDER. To assess the overall quality of ADR discovery by our method, we computed the Spearman rank correlation (ρ) between the 2 lists of ADRs for each drug, 1 ordered by QLRS and 1 by the number of AERS reports.

The AERS data are complicated by the fact that multiple reports can be submitted to the FDA for the same case, and that reports can pertain to side effects of the drug, the underlying disease, or another drug taken concurrently [29]. Therefore, ADR prevalence according to AERS should be considered a noisy reference. We employed several approaches to computing the correlation. First, we used the raw report counts in AERS, and denote the correlation between these and QLRS by ρ_1_. We also used the AERS data to compute 2 regularized measures of disproportionality that are commonly employed for analyzing adverse side effect reports. Specifically, we used the empirical Bayes geometric mean (EBGM) [30,31], and denote the correlation between EBGM computed for AERS and the QLRS by ρ_2_. Finally, the correlation between the information component (IC) [32] computed for AERS and QLRS is denoted by ρ_3_.

We hypothesized that some ADRs are more likely to be reported to the FDA, whereas others tend to be self-addressed by patients through online research. Consequently, if our method was to discover previously unknown ADRs, the correlation can never be perfect. Therefore, we first analyzed the commonalities among the ADRs we discovered and those already known. Then, we analyzed the properties of the newly discovered ADRs.

To focus on the ADRs identified by both our method and the AERS data, we removed the 5 symptoms that most reduced the value of 1 of the metrics, ρ_1_, using a greedy selection process. We call the removed symptoms the most discordant ADRs (MDADRs). Specifically, we iteratively identified and removed the ADR that most reduced the Spearman rank correlation between the AERS counts and the QLRS ranking of ADRs. An alternative method of removing discordant ADRs would focus on reaching statistically significant values of ρ_1_. However, we chose to use a fixed number to facilitate the analysis of MDADRs, as performed subsequently.

### Identification of Adverse Drug Reactions for Multiple Drugs

Some individuals are prescribed multiple drugs to be taken simultaneously. The interaction between these drugs may give rise to specific ADRs that are not present (or are present at different severity) if each drug is taken individually. Thus, in the following we show how our method can be used to identify ADRs that are associated with taking pairs of drugs. Our method attempts to remove the ADRs attributed to individual drugs so as to identify those ADRs that arise from the combination thereof.

For each pair of drugs that were analyzed, we identified their characteristic ADRs caused by the interaction by discounting the probability of the ADRs arising from each of the individual drugs. This was done by subtracting the contribution of ADRs of the individual drugs as predicted by a linear regression model. We hypothesized that the ADRs observed in patients who only take 1 of the drugs, will appear at a similar ratio for the patients who take both drugs. However, new ADRs that are caused by the interaction of the 2 drugs will not be reliably predicted by modeling each drug separately and will, therefore, appear at a substantially different ratio than the prediction.

For each pair of drugs, we identified 3 disjoint groups of users: the first 2 groups are those who searched for only 1 of the 2 drugs, and the third group searched for both. For the first 2 groups, we counted the number of times each ADR was searched for before and after Day Zero. For the third group (ie, users who searched for both drugs), we defined Day Zero as max(date-first-search(drug_i_), date-first-search(drug_j_)), where date-first-search() is the earliest date on which the user searched for a given drug. We denote these numbers (before/after Day Zero) for the i-th ADR in population p by n^p^_i,b_ (n^p^_i,a_). Next, we defined the ratio of change in the ADR prevalence (after the commencement of treatment with the second drug) as n^p^_i,a_/(n^p^_i,b_ + n^p^_i,a_). Finally, we built a regression model to predict the probability of change in the third population (patients taking both drugs) given the corresponding values in the first 2 populations. This regression model effectively discounts the effect of the ADRs caused by each drug separately. We also identified MDADRs for pairs of drugs in a similar way as for individual drugs.

## Results

We counted the number of times each drug appeared in AERS, and found it to be highly correlated with the number of online searches for that drug. For the drugs listed in Table 2, ρ = 0.66 (*P*=.002; n=20). The correlation becomes even more pronounced for pairs of drugs, ρ = 0.73 (*P*<.001; n=380). The correlation between the sales figures (as represented by the number of prescriptions sold) and the number of Web searches is *R* ^*2*^=0.26 (*P*=.005). A linear model that uses both the number of AERS reports and the sales figures to predict Web search volume yields a *R* ^*2*^ 2.4% greater than the one using only AERS reports. We believe these findings mean that the search volume is more indicative of the prevalence of ADRs rather than actual sales. Thus, the popularity of a drug in Web queries is highly representative of its appearance in AERS, suggesting that Web queries are strongly reflective of real-world phenomena.

As noted in the Methods section, we assessed the overall quality of ADR discovery by our method by computing the Spearman rank correlation coefficient between 2 lists of ADRs for each drug, 1 ordered by QLRS and 1 by the number of AERS reports. Table 2 reports the values of ρ_1_ after removing 5 MDADRs for each drug. QLRS predictions are relatively highly correlated with the AERS counts, and the correlation is statistically significant (*P*<.05) for 12 of the 20 drugs using the Olkin-Pratt (DSL) fixed-effect meta-analytical approach [33] (*P*<.001; n=20). Positive correlation was not found in only 1 of the drugs (Singulair). Interestingly, removing 15 MDADRs for this drug (instead of 5) resulted in a statistically significant correlation of ρ_1_ = 0.48 (*P*=.02), suggesting a particularly high discrepancy between the prevalence of ADRs as predicted by QLRS and as registered in AERS for this drug.

We also note that although most of the observed correlation values are significant, they are far from indicating perfect correlation. This is to be expected because the correlation would only have been perfect if our method were exactly rediscovering the known ADRs. However, as we discovered previously unknown ADRs, we obviously achieved an imperfect match to the list of known ones in AERS. In the following section, we analyze the differences between the known ADRs and those identified by our method. Additionally, there is a small negative correlation (ρ=–0.22, *P*=.02) between the number of users who queried for a drug and ρ_1_. This demonstrates that higher correlations are obtained when more data are available, and is an additional cause for the imperfect correlations.

Statistically significant correlations with EBGM and IC were also found (see Multimedia Appendix 1), and the meta-analysis is statistically significant (*P*<.001; n=100). However, EBGM and IC are measures designed to enhance the detection of ADRs that are especially prevalent in a given drug under study compared with all other drugs. At the same time, raw AERS counts (used for the computation of ρ_1_) are more likely to be associated with the appearance of an ADR regardless of any other drug. This explains the higher correlation we observed of QLRS with the raw AERS counts (ρ_1_) than with EBGM and IC (ρ_2_ and ρ_3_, respectively).

SIDER [27] contains information on ADRs extracted from public documents and package inserts. Because of regulatory and legal requirements, it is overly inclusive in its listings, which makes it a noisy reference as well. The SIDER data are essentially binary, without relative frequency or absolute counts, which makes the previous correlation analysis inapplicable. We used SIDER to assess the accuracy of QLRS by computing the area under the curve (AUC) [34] of a receiver operating characteristic (ROC) curve and the *F* score [35], taking as positive examples all the ADRs listed in SIDER for the drug. AUC measures the method’s ability to correctly identify known ADRs, whereas the *F* score simultaneously considers precision and recall. Only 8 of the 20 drugs we analyzed appeared in SIDER, and the corresponding accuracies are reported in Table 3 (after removing MDADRs). The results suggest that our method is able to reconstruct known ADRs, as measured with AUC and the *F* score.

**Table 2:** Spearman rank correlation (ρ) between query log reaction score (QLRS) and the number of adverse drug reaction (ADR) reports in the Adverse Event Reporting System (AERS), with the most discordant ADRs (MDADRs) removed.

Drug	ρ_1_	*P* value ^a^	MDADRs^b^
Advair	0.28		Anxiety, apnea,^c^ chest pain, cough, weight gain^c^
Aranesp	0.30		Asthenia, back ache, back pain, edemac
Diovan	0.34		Chest pain, cramp,^c^ sleepy,^c^ wound
Effexor	0.54	<.001	Nausea, phobia,^c^ sleepy,^c^ weight gain^c^
Enbrel	0.39	.02	Back pain, cough, diarrhea, fever, weight gain^c^
Lipitor	0.54	<.001	Asthenia, constipation, diarrhea, dizziness, nausea
Mabthera	0.38	.01	Chest pain, fever, headache, malaise, wound^c^
Nexium	0.45	.008	Abdominal pain, tired,^c^ weak,^c^ weight gain^c^
Norvasc	0.34		Apnea,^c^ constipation, cramp,^c^ tired,^c^ weight loss^c^
Pantoloc	0.49	.001	Chest pain, fever, headache, malaise, nausea
Pantozol	0.51	.006	Chest pain, fever, headache, malaise, nausea
Plavix	0.25		Back pain, chest pain, cough, paresthesia
Protonix	0.25		Abdominal pain, diarrhea, nausea, vomit^c^
Remicade	0.37	.04	Chest pain, fever, infertility,^c^ paresthesia, rash
Risperdal	0.40	.02	Diarrhea, headache, insomnia, weight gain^c^
Rituxan	0.23		Abdominal pain, diarrhea, paresthesia, weak^c^
Seretide	0.41	0.004	Chest pain, dyspnea,^c^ headache, malaise, nausea
Seroquel	0.48	0.004	Apnea,^c^ dizziness, headache, weight gain^c^
Singulair	–0.06		Apnea,^c^ dizziness, insomnia, tired^c^
Zyprexa	0.61	0.002	Constipation, diarrhea, nausea, paresthesia, somnolence

^a^ *P* values are provided for statistically significant correlations (n=45).

^b^Unless otherwise indicated, MDADRs are those prominent in AERS.

^c^MDADRs emphasized in QLRS.

**Table 3:** Accuracy of adverse drug reaction (ADR) identification by using QLRS, tested against the SIDER dataset with most discordant ADRs (MDADRs) removed.

Drug	*F* score (df)	AUC
Advair	0.77	0.67
Diovan	0.43	0.71
Effexor	0.94	0.67
Lipitor	0.76	0.7
Pantoloc	0.44	0.57
Pantozol	0.44	0.64
Plavix	0.55	0.59
Singulair	0.52	0.64

### Most Discordant Adverse Drug Reactions

Analyzing the MDADRs revealed characteristic differences among the known ADRs (registered in AERS and SIDER) and those identified by our method. The ADRs identified as most discordant are not random; instead, they belong to 1 of the following 2 classes, as shown in Table 4. The first class includes ADRs that are readily recognized by patients and medical professionals because of their acuteness and fast onset. The other class includes later onset, less acute ADRs, which are more difficult to identify using self-reporting methods.

As noted previously, upon removing as few as 5 MDADRs, the correlation between QLRS and AERS counts (ρ_1_) frequently becomes statistically significant. Conversely, removing a random subset of 5 symptoms only results in a negligible, statistically insignificant change in the correlation.

Although the MDADRs were identified separately for each drug, they were highly consistent across drugs. Of the 32 MDADRs we identified overall, 22 were chosen for more than 1 drug (mean 3.1, SD 2.3). Significantly, these ADRs were always overemphasized either in the query log or in AERS, but never in both (for different drugs). The likelihood of such behavior at random is smaller than 1:105. A typical example is the ADR nausea, which appeared at a far higher rank (ie, more prevalent) in the AERS dataset than in the QLRS ranking for 7 out of the 20 drugs, and was never found at a rank below that of QLRS for the other drugs.

**Figure 1:**
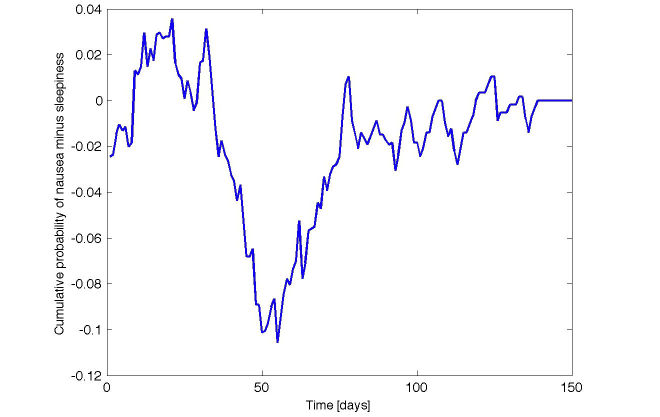
Temporal behavior of adverse drug reactions (ADRs). The difference between the cumulative probabilities of the ADRs “nausea” and “sleepiness” for the drug Effexor. The ADR highly ranked by QLRS (sleepiness) has a much later onset (45-75 days).

Most importantly, MDADRs that are prominent in queries and in AERS have notable differences in their temporal behavior. As an illustrative example, we used the query log to compute the cumulative density functions (CDFs) over time for 2 MDADRs for the drug Effexor, 1 overemphasized in AERS (nausea) and 1 overemphasized by QLRS (sleepiness). Figure 1 shows the difference between the CDFs of the 2 MDADRs, starting from the time the drug is first searched for (day 0). As Figure 1 demonstrates, each of these 2 MDADRs is more likely to occur in a different time range. Observe that the symptom prominent in AERS (nausea) is usually searched for shortly after the first query about the drug (ie, several days after Day Zero), when it is much more likely than the other symptom. In contrast, the symptom ranked highly by QLRS (sleepiness) appears much more prominently 45 to 75 days after the commencement of treatment, when the likelihood of nausea drops significantly.

We measured the difference in the time of onset (defined as the number of days between the first search for the drug and the first search for the ADR in the query log) for the MDADRs that were overemphasized by QLRS and in the AERS data. Averaged over all the drugs, the difference was 7.3 days (2-sided Wilcoxon signed rank test [36], *P*=.01; n=15). Based on these findings, we conclude that ADRs are more likely to be reported to the regulatory authorities if they appear shortly after commencing the treatment (as it might be easier for patients and caregivers to link the ADRs to the treatment), and that are serious enough to warrant reporting. Conversely, ADRs identified by our method usually appear much later after the beginning of treatment; hence, their possible association to the drug is often overlooked.

Thus, the MDADRs overemphasized by QLRS represent an interesting class of reactions that are harder to discover using traditional methods.

**Table 4:** Most discordant adverse drug reactions (MDADRs) identified (out of the 20 drugs analyzed).

MDADRs		n^a^
**Overrepresented in AERS**		
	Abdominal pain	3
	Asthenia	2
	Back pain	3
	Chest pain	8
	Constipation	3
	Cough	3
	Diarrhea	8
	Dizziness	3
	Fever	5
	Headache	6
	Insomnia	2
	Malaise	4
	Nausea	7
	Paresthesia	4
**Overrepresented in the query logs**		
	Apnea	4
	Cramps	2
	Sleepy	2
	Tired	3
	Weak	2
	Weight gain	6
	Wound	2

^a^ Number of drugs in which MDADR appeared.

### Correlation Between Query Log Reaction Scores of Similar Drugs

Several of the drugs we investigated are different brands of essentially the same drug. All other things being equal, we expected that patients taking 2 different brand versions of the same drug would experience similar ADRs. To evaluate this conjecture, we conducted 2 evaluations. First, we measured the Spearman correlation between the QLRS of ADRs for the multiple brand versions of the same drug. Second, we evaluated the correlation between the QLRS and AERS counts after aggregating the chi-square contingency tables for drugs that have the same generic names.

The intradrug correlation (different brands of the same drug) was, on average, 0.42 compared to 0.23 for all the other pairs of drugs (*P*=.03, 1-sided rank sum test). Thus, although the ADRs are somewhat different among the near-identical drugs, the correlation is statistically significantly higher than that observed for random pairings of drugs. The imperfect correlation can be explained by several factors. First, different manufacturers may produce drugs with slight variations in inactive ingredients, coloring agents, and fillers. The change in fillers in the drug Eltroxin in Israel has been associated with a large number of patients experiencing major side effects, including changes in heart rate, dizziness, and difficulties in breathing [37]. Furthermore, there could be demographic differences between the populations taking those drugs. For example, different brand versions may be prescribed in different markets or different geographical regions. Finally, although many health care providers report that they do not employ special monitoring after switching from brand names to generic drugs, some have encountered specific ADRs caused by switching [38]. Thus, there are known differences in ADRs caused by different versions of similar drugs.

Nonetheless, the relatively high correlation between the ADRs of similar drugs provides additional supporting evidence that the ADRs discovered by QLRS are a genuine reflection of actual patient experiences.

By using RxNorm [39] we identified 30 drugs that are different brand versions of 14 generic drugs, out of the 100 drugs that we analyzed (see Multimedia Appendix 1). For example, Procrit and Eprex are 2 brand versions of the generic drug erythropoietin. This allowed us to focus the analysis on the generic component of the drug rather than the specific brand names, by computing an aggregated contingency table for all brand name versions of the same generic drug before computing the QLRS (the AERS counts were aggregated similarly).

The resulting correlations are reported in Table 5. The values of ρ_1_ aggregated over all the brand names of the same generic drug are significantly higher than those for individual brand names (on average, aggregated ρ_1_ = 0.62 compared to 0.35 for individual brand names). In all 14 cases, statistically significant correlations between the QLRS and AERS counts were found. MDADRs found in the aggregated data were the same as those identified in the brand name versions of the drug in 79% of the cases.

**Table 5:** Spearman rank correlation (ρ) between the adverse drug reports (ADRs) of generic drugs as identified by query log reaction score (QLRS) and by the number of reports in the Adverse Event Reporting System (AERS), with most discordant ADRs (MDADRs) removed according to the raw report counts in AERS. The QLRS and AERS counts for the generic drugs were computed by aggregating over multiple brand names of the same generic drug. For statistically significant correlations, *P* values are provided (n=45).

Brand names	Generic name	Averaged ρ_1_ of individual brand names	ρ_1_ aggregated over all the brand names of the same generic drug	*P* value
Procrit, Eprex	Erythropoietin	0.35	0.72	<.001
Neulasta, Neupogen	Filgrastim	0.43	0.54	.003
Lantus, Humalog	Insulin analog	0.43	0.60	<.001
Avonex, Rebif	Interferon beta-1a	0.60	0.83	<.001
AcipHex, Pariet	Rabeprazole	0.21	0.53	.002
Protonix, Pantozol, Pantoloc	Pantoprazole	0.42	0.67	<.001
TriCor, Lipanthyl	Fenofibrate	0.40	0.70	<.001
Rituxan, MabThera	Rituximab	0.31	0.52	.003
Advair, Flovent	Fluticasone	0.31	0.64	<.001
Cozaar, Hyzaar	Losartan	0.42	0.56	<.001
Losec, Prilosec	Omeprazole	0.27	0.65	<.001
Paxil, Seroxat	Paroxetine	0.13	0.44	.002
Avandamet, Avandaryl, Avandia	Rosiglitazone	0.38	0.67	<.001
Imigran, Imitrex	Sumatriptan	0.26	0.60	<.001

We attribute these findings to several reasons. First, by aggregating different brand name versions of a drug we focus on the active ingredient of the drug, which is more likely to be reported in AERS by medical personnel. Second, averaging these additional observations over multiple drugs reduces the amount of noise in the data, and thus increases the correlation with AERS. Finally, analyzing the active ingredient reduces the effect of individual manufacturing procedures and components, and thus focuses the analysis on a simpler set of chemical components, which may have a smaller set of ADRs.

### Adverse Reactions to Multiple Drugs

Some ADRs occurred only when 2 drugs were taken concurrently or in close temporal proximity. These ADRs can be especially difficult to detect because they occur infrequently, and only in a population that takes both drugs. To this end, we extended our method to identify ADRs of pairs of drugs.

The correlation between QLRS rankings and AERS (raw counts, EBGM, and IC), for the 10 most common pairs of drugs, is shown in Table 6. These correlations are lower than those for individual drugs, but are still statistically significant using the Olkin-Pratt (DSL) fixed-effect meta-analytical approach [33] (*P*<.001; n=10). We believe this result is noteworthy because it may be more difficult to include in a clinical trial those patients who take multiple drugs concurrently.

**Table 6:** Spearman rank correlation (ρ) between the adverse drug reactions (ADRs) of pairs of drugs identified by query log reaction score (QLRS) and by the number of reports in the Adverse Event Reporting System (AERS), with most discordant ADRs (MDADRs) removed according to the raw report counts in AERS.

Drug 1	Drug 2	ρ_1_	ρ_2_	ρ_3_
Risperdal	Seroquel	0.27	–0.08	–0.16
Effexor	Advair	0.28	–0.11	0.04
Zyprexa	Seroquel	0.1	0.15	–0.05
Advair	Lipitor	–0.21	0.05	0.02
Plavix	Lipitor	0.3	0.29	0.24
Lipitor	Effexor	0.14	–0.18	–0.02
Advair	Plavix	0.14	0.15	0.05
Nexium	Plavix	0.19	0.11	0.33
Seroquel	Effexor	0.32	0.15	0.11
Lipitor	Nexium	0.12	0.07	0.14

Based on these findings, we believe our method can also be applicable to combination products (ie, drugs that contain 2 or more active substances), if each of the active substances is marketed also as a separate drug in similar doses. We plan to extend our method to combination products in our future work, and intend to investigate whether the correlation can be increased, for example, by using nonlinear correlation measures.

In their recent work, White et al [40] used search logs to study the side effects of 1 specific drug pair, paroxetine and pravastatin, whose interaction was reported to cause hyperglycemia. Their finding confirms the utility of search logs in identifying drug interactions, which were later validated by the FDA. However, there are several key differences between their study and ours. First, the method proposed by White et al performs a direct count of symptoms, thus not taking into account seasonal and other effects handled by the QLRS method. Second, to identify queries that are indicative of hyperglycemia, White et al constructed a list of hyperglycemia-related terms manually by reviewing the relevant medical literature. In contrast, we map user queries to medical terminology in an automated way, building on query expansion methods developed in the field of information retrieval. Finally, whereas White et al only analyzed 1 particular condition (hyperglycemia) and 1 specific pair of drugs (paroxetine and pravastatin), our study was conducted on a substantially larger scale. Specifically, we automatically mined side effects of 100 top-selling drugs and their combinations, and side effects of vaccines.

## Discussion

### Principal Findings

Clinical trials of pharmaceutical drugs are limited in their extent owing to their prohibitively high cost and insufficient diversity among participants. On the other hand, voluntary reporting of ADRs by patients and health care professionals is limited because of the extra effort required, and because of the difficulty of linking the ADRs to the drug that caused them (especially when these ADRs have a late onset or are caused by multidrug treatments). We propose a novel, low-cost method for discovering adverse drug reactions from aggregated Web search data of large populations of Internet users. We demonstrated that our method allows analyzing the ADRs of drugs and vaccines in dramatically larger populations than typical clinical trials, and can assist in identifying ADRs that have so far eluded discovery by the existing mechanisms.

We believe our method constitutes a new, complementary approach to pharmacovigilance, because of its computational efficiency and access to vastly larger and more diverse populations. There are multiple avenues for future work. The effectiveness of our method can be validated by analyzing medical records (eg, OMOP), or by assessing its ability to predict changes in safety labels by regulatory authorities. It would also be interesting to compare the QLRS to those derived from the analysis of social media. Finally, a validation of MDADRs through clinical trials would be of significant value to validate our method. Specifically, we propose to test the MDADRs found by our method, which are underemphasized in current ADR databases (eg, AERS), in a clinical setting or through phase 4 trials. Such trials should be prioritized by the severity, volume of searches, and uniqueness of the ADRs discovered. Once verified, these MDADRs will become an important addition to the list of known ADRs of which patients are informed. Finally, quantifying the strength of the protopathic bias (if any) in our data would serve to strengthen the validity of ADRs discovered by our method.

Our work falls within the domain of infodemiology; that is, the study of Internet media to inform public health and policy [15]. Much previous work in this area has centered on detection and characterization of transient events (ie, disease outbreaks [15] and special events [40]) and the analysis of the kinds of information available to users [41,42]. Our paper is novel in that it makes use of search engine queries to identify transient events at the individual level and, more importantly, to discover associations between events [15] that eluded detection by the patients themselves or their health practitioners.

### Limitations

The main drawback of relying on Web search data is that it is inherently noisy. It is often impossible to ascertain whether a person searching for drugs and ADRs is doing so out of curiosity, or conducting research for himself, a relative, or even for a patient. Admittedly, Internet users comprise a biased sample of the population, and so the ADRs discovered may not be fully representative of the entire population. Nonetheless, our results suggest that the sheer size of the data alleviates these concerns, and the proposed method is able to identify adverse effects of drugs that are not captured by existing surveillance mechanisms.

Another limitation of this study is using a restricted set of symptoms expanded through the use of synonyms. Although a larger dictionary would have allowed identification of additional (and possibly rarer) ADRs, our focus on more common symptoms is likely to lead to better identification of the more common concerns to patients. Future work will focus on professionally used term dictionaries to focus on more knowledgeable patients and health providers. Another way to strengthen our results is the use of non-English search data, which will increase the volume of data (and the size of the observed population); thus, enabling the analysis of less frequent drugs and ADRs.

Finally, although this work is based on data from a large Internet search engine, it does not cover the entire population. However, privacy concerns preclude conducting our analysis across search engines, as the latter never share information about their users. Nevertheless, given the sheer number of users whose data was analyzed in the study (176 million, which is especially notable compared to most other pharmacovigilance studies), we believe our findings are still significant. It should also be emphasized that QLRS discovers ADRs via aggregating queries across multiple users and query sessions. Consequently, the output of our method comes in the form of a list of newly discovered ADRs for each drug, and does not include any private, personal, or user-specific data whatsoever.

### Conclusions

Our approach is mostly language-independent except for the initial list of symptoms [20], and obtaining the latter from non-English versions of Wikipedia will allow one to apply the method to additional languages, markets, and populations. Extending the coverage is particularly important for studying rare events, such as ADRs in patients who take many prescription drugs. Computational pharmacovigilance, which uses observational data such as Web search query logs, is complementary to the existing data collection mechanisms, and the ADRs it identifies should be considered as candidates for further investigation.

## Multimedia Appendix 1

Identification of adverse drug reactions in vaccines.

## Abbreviations

ADR: adverse drug reaction
AERS: Adverse Event Reporting System
AUC: area under the curve
CDF: cumulative density functions
EBGM: empirical Bayes geometric mean
FDA: Food and Drug Administration
IC: information component
MDADR: most discordant adverse drug reactions
MHRA: Medicines and Healthcare products Regulatory Agency
QLRS: query log reaction score
RADAR: Research on Adverse Drug Events and Reports
ROC: receiver operating curve
SIDER: Side Effect Resource
VAERS: Vaccine Adverse Event Reporting System
WHO: World Health Organization
