# Supplementary material for: Postmarket Drug Surveillance Without Trial Costs: Discovery of Adverse Drug Reactions Through Large-Scale Analysis of Web Search Queries
Source: J Med Internet Res. 2013 Jun 18;15(6):e124. doi: 10.2196/jmir.2614 (PMC3713931; doi:10.2196/jmir.2614)
Supplement: Supplementary file 1 [file jmir_v15i6e124_app1.pdf]

Appendix

Identification of Adverse Drug Reactions in Vaccines

Another class of commonly prescribed drugs is vaccines, which are administered mostly to children, but also to adults. Though serious ADRs of vaccines are extremely rare, less severe reactions are known to occur. The FDA keeps track of such adverse reactions using a database similar to AERS, called the Vaccine Adverse Event Reporting System (VAERS). We used VAERS as the reference dataset for identification of ADRs in vaccines. VAERS data was downloaded from the FDA VAERS website, and included reports submitted between January 2004 and December 2009.

Using our methodology, we compared the ADRs identified by QLRS with those reported to VAERS, for the 11 vaccines most commonly mentioned in the queries (see Table S3). In general, correlations between ADRs identified by our method and those registered in VAERS are high. Indeed, for 10 of the 11 vaccines the Spearman correlation was statistically significant (after removal of MDADRs). The MDADRs emphasized in VAERS were “diarrhea", “fever", “nausea", and “headache". On the other hand, the most prominent MDADRs according to QLRS were “tiredness" and “weight loss".

These findings demonstrate that the proposed approach for identifying ADRs of pharmaceutical drugs can also be applied to vaccines, which are usually administered only a limited number of times to each patient.

Table S1. Spearman's rank correlation between QLRS and the number of ADR reports in AERS, EBGM, and IC (with MDADRs removed according to the number of reports in AERS) for the top 100 drugs. For statistically significant correlations, p-values are provided in parentheses (n=45).

**Drug**	**1**	**2**	**3**
Abilify	0.05	0.04	0.04
Aciphex	-0.02	-0.19	-0.19
Actonel	0.05	0.18	0.18
Actos	0.16	0.3	0.3
Advair	0.28	0.14	0.14
Aranesp	0.32	0.34 (0.04)	0.34 (0.04)
Atacand	0.39 (0.02)	0.09	0.09
Augmentin	0.27	0.17	0.17
Avandamet	0.39 (0.01)	0.4 (0.009)	0.4 (0.009)
Avandaryl	0.4 (0.009)	0.33 (0.03)	0.33 (0.03)
Avandia	0.35 (0.04)	0.32	0.32
Avastin	-0.01	-0.18	-0.18
Avonex	0.48 (0.002)	0.47 (0.003)	0.47 (0.003)
Betaseron	0.51 (0.0007)	0.47 (0.002)	0.47 (0.002)
Celebrex	0.16	0.08	0.08
Cellcept	0.61 ($<10^{-5}$)	0.68 ($<10^{-5}$)	0.68 ($<10^{-5}$)
Cipralex	0.74 ($<10^{-5}$)	0.66 ($<10^{-5}$)	0.66 ($<10^{-5}$)
Clexane	0.72 ($<10^{-5}$)	0.69 ($<10^{-5}$)	0.69 ($<10^{-5}$)
Copaxone	0.26	0.13	0.13
Coreg	0.22	0.09	0.09
Cozaar	0.48 (0.003)	0.21	0.21
Crestor	0.26	-0.07	-0.07
Cymbalta	0.42 (0.01)	0.14	0.14
Delix	0.35 (0.02)	0.34 (0.03)	0.34 (0.03)
Depakote	0.42 (0.01)	0.11	0.11
Detrol	0.71 ($<10^{-5}$)	0.33 (0.03)	0.33 (0.03)
Diovan	0.34	0.1	0.1
Duragesic	0.46 (0.002)	0.28	0.28
Effexor	0.54 (0.0008)	0.23	0.23

Eloxatin	0.49 (0.0008)	0.44 (0.004)	0.44 (0.004)
Enbrel	0.39 (0.02)	0.32	0.32
Eprex	0.33 (0.03)	0.28	0.28
Erbitux	0.31 (0.04)	0.33 (0.03)	0.33 (0.03)
Evista	0.39 (0.02)	0.34 (0.04)	0.34 (0.04)
Flomax	-0.08	-0.33	-0.33
Flovent	0.34 (0.05)	0.21	0.21
Floxin	0.08	0.1	0.1
Fosamax	-0.02	0.08	0.08
Gemzar	0.5 (0.0008)	0.49 (0.001)	0.49 (0.001)
Gleevec	0.18	0.08	0.08
Harnal	0.38 (0.01)	0.29	0.29
Herceptin	0.77 ($<10^{-5}$)	0.65 ($<10^{-5}$)	0.65 ($<10^{-5}$)
Humalog	0.48 (0.002)	0.48 (0.003)	0.48 (0.003)
Humira	0.58 (0.0001)	0.51 (0.001)	0.51 (0.001)
Hyzaar	0.36 (0.03)	0.15	0.15
Imigran	0.33 (0.03)	0.2	0.2
Imitrex	0.18	0.04	0.04
Kaletra	0.58 ($<10^{-5}$)	0.45 (0.003)	0.45 (0.003)
Lamictal	0.27	-0.18	-0.18
Lantus	0.38 (0.02)	0.26	0.26
Levaquin	0.61 (0.0001)	0.29	0.29
Lexapro	0.12	0.13	0.13
Lipanthyl	0.34 (0.03)	0.34 (0.03)	0.34 (0.03)
Lipitor	0.54 (0.0009)	0.2	0.2
Losec	0.3	0.07	0.07
Lotrel	0.42 (0.01)	0.19	0.19
Lovenox	0.35 (0.04)	-0.03	-0.03
Lupron	0.08	-0.2	-0.2
Lyrica	-0.12	-0.24	-0.24
Mabthera	0.38 (0.01)	0.3	0.3
Micardis	0.24	0.3	0.3
Neorecormon	0.34 (0.03)	0.23	0.23
Neulasta	0.53 (0.0003)	0.42 (0.006)	0.42 (0.006)
Neupogen	0.33 (0.04)	0.33 (0.04)	0.33 (0.04)
Nexium	0.45 (0.008)	0.12	0.12

Norvasc	0.34	0.14	0.14
Olmetec	0.51 (0.0007)	0.4 (0.01)	0.4 (0.01)
Pantoloc	0.49 (0.001)	0.46 (0.002)	0.46 (0.002)
Pantozol	0.51 (0.0006)	0.45 (0.003)	0.45 (0.003)
Pariet	0.43 (0.004)	0.38 (0.01)	0.38 (0.01)
Paxil	-0.05	-0.1	-0.1
Pegasys	0.49 (0.001)	0.39 (0.01)	0.39 (0.01)
Plavix	0.25	-0.04	-0.04
Pravachol	0.21	-0.1	-0.1
Premarin	0.34	0.01	0.01
Premphase	0.4 (0.01)	0.31 (0.04)	0.31 (0.04)
Prempro	0.62 ($<10^{-5}$)	0.5 (0.001)	0.47 (0.003)
Prevacid	0.29	-0.05	-0.05
Prevnar	0.6 ($<10^{-5}$)	0.6 ($<10^{-5}$)	0.6 ($<10^{-5}$)
Prilosec	0.23	0.06	0.06
Procrit	0.37 (0.03)	0.43 (0.01)	0.43 (0.01)
Prograf	0.61 ($<10^{-5}$)	0.62 ($<10^{-5}$)	0.62 ($<10^{-5}$)
Protonix	0.25	0.04	0.04
Pulmicort	0.53 (0.0006)	0.49 (0.002)	0.49 (0.002)
Rebif	0.71 ($<10^{-5}$)	0.61 ($<10^{-5}$)	0.61 ($<10^{-5}$)
Remicade	0.36 (0.04)	0.41 (0.02)	0.41 (0.02)
Risperdal	0.4 (0.02)	0.01	0.01
Rituxan	0.23	0.3	0.3
Seretide	0.58 (0.00006)	0.25	0.23
Seroquel	0.48 (0.003)	-0.11	-0.11
Seroxat	0.3	0.24	0.24
Singulair	-0.06	-0.13	-0.13
Spiriva	0.46 (0.006)	0.33	0.33
Symbicort	0.27	0.21	0.21
Synagis	0.63 ($<10^{-5}$)	0.65 (0.000004)	0.65 (0.000004)
Tamiflu	0.69 ($<10^{-5}$)	0.69 (0.000001)	0.69 (0.000001)
Taxotere	0.52 (0.0004)	0.38 (0.02)	0.38 (0.02)

Topamax	0.36 (0.04)	0.26	0.26
Toprol	0.51 (0.002)	0.14	0.14
Tricor	0.45 (0.006)	0.37 (0.03)	0.37 (0.03)
Tritace	-0.08	-0.08	-0.08
Truvada	0.45 (0.003)	0.59 (0.00004)	0.59 (0.00004)
Valtrex	0.12	0.02	0.02
Viagra	0.28	-0.07	-0.07
Vytorin	0.39 (0.02)	0.41 (0.01)	0.41 (0.01)
Wellbutrin	0.08	0.11	0.11
Xalatan	0.3	0.3	0.3
Zetia	0.26	0.06	0.06
Zocor	0.09	-0.04	-0.04
Zofran	0.1	0.15	0.15
Zoladex	0.46 (0.002)	0.33 (0.03)	0.33 (0.03)
Zoloft	0.12	0.19	0.19
Zometa	0.33 (0.04)	0.27	0.27
Zyprexa	0.61 (0.0002)	0.11	0.11

Table S2. Accuracy of ADR identification using QLRS for the top 100 drugs, tested against the SIDER dataset (MDADRs removed).

Drug	F-measure	AUC
Abilify	0.79	0.86
Aciphex	0.46	0.86
Actonel	0.64	0.81
Advair	0.78	0.7
Atacand	0.87	0.76
Avandia	0.83	0.55
Blopress	0.50	0.39
Celebrex	0.61	0.79
Coreg	0.79	0.82
Cozaar	0.74	0.8
Crestor	0.69	0.75
Cymbalta	0.72	0.81
Delix	0.57	0.33
Diovan	0.71	0.78
Duragesic	0.67	0.5
Effexor	0.67	0.9
Eloxatin	0.71	0.6
Eloxatine	0.50	0.44
Evista	0.88	0.6
Flomax	0.62	0.64
Flovent	0.67	0.6
Gemzar	0.69	0.5
Gleevec	0.68	0.61
Harnal	0.60	0.29
Hyzaar	0.69	0.75

Imigran	0.55	0.42
Imitrex	0.47	0.86
Lamictal	0.61	0.9
Lipanthyl	0.56	0.33
Lipitor	0.70	0.75
Losec	0.72	0.62
Lotrel	0.63	0.75
Lyrica	0.51	0.92
Micardis	0.55	0.71
Pantoloc	0.57	0.44
Pantozol	0.64	0.44
Pariet	0.65	0.46
Paxil	0.73	0.83
Plavix	0.59	0.55
Pravachol	0.66	0.65
Prilosec	0.58	0.9
Prograf	0.79	0.83
Protonix	0.73	0.9
Pulmicort	0.88	0.8
Risperdal	0.88	0.94
Seloken	0.50	0.44
Seroquel	0.66	0.89
Seroxat	0.54	0.44
Singulair	0.64	0.52
Spiriva	0.90	0.7
Symbicort	0.61	0.6
Takepron	0.50	0.58

Taxotere	0.74	0.67
Toprol	0.76	0.85
Tricor	0.72	0.78
Tritace	0.57	0.33
Viagra	0.79	0.75
Wellbutr<br><br>in	0.77	0.91
Zetia	0.94	0.8
Zocor	0.55	0.57
Zofran	0.48	0.47
Zoloft	0.57	0.81

Table S3. Spearman's rank correlation between QLRS and the number of ADR reports in VAERS (with MDADRs removed according to the raw report counts in VAERS). For statistically significant correlations, p-values are provided in parentheses. Also shown are MDADRs for each drug and the main indications for the drug. MDADRs shown in bold are those emphasized in QLRS, and those in regular type are prominent in VAERS.

**Vaccine**	**Indications**	**1**	**2**	**3**	**MDADRs**
DPT	Diphtheria, pertussis, tetanus	0.46 (0.01)	0.44 (0.01)	0.36 (0.04)	**Alopecia**, **Apnea**, **Dry mouth**, **Depression**, Edema
Hepatitis	Hepatitis	0.41 (0.02)	0.07	0.21	**Apnea**, **Depression**, Nausea, **Weight loss**
HPV	Human papiloma virus	0.33 (0.05)	0.26	0.15	Headache, **Infertility**, **Miscarriage**, **Tired**
IPV	Polio	0.51 (0.003)	0.48 (0.005)	0.41 (0.02)	Bleeding, Malaise, **Weight loss**, Urticaria
MMR	Measles, mumps, rubella	0.45 (0.01)	0.40 (0.02)	0.50 (0.002)	Edema, **Sleepy**, **Tired**, **Weight loss**
OPV	Polio	0.69 ($<10^{-5}$)	0.58 (10^{-4})	0.72 ($<10^{-5}$)	**Bloody show**, **Chest pain**, **Hirsutism**, **Tired**
Polio	Polio	0.51	0.40 (0.02)	0.41 (0.01)	**Depression** , **Itch**,

		(0.002)			**Phobia**, **Wound**
Smallpox	Smallpox	0.44 (0.01)	0.42 (0.02)	0.31	Diarrhea, Fever, Headache, Paresthesia, **Tired**
Typhoid	Typhoid fever	0.33 (0.05)	0.41 (0.01)	0.33	Diarrhea, Fever, **Weight loss**, **Xerostomia**
Varicella	Chickenpox	0.18	0.10	0.10	Fever, **Miscarriage**, **Tired**, Urticaria
Yellow fever	Yellow fever	0.46 (0.005)	0.37 (0.02)	0.55 ($5*10^{-4}$)	**Drug overdose**, Nausea, **Tired**, **Weight loss**
